# Fueling the sentinels: organelle remodeling and metabolic adaptation in tissue-resident memory T cell persistence

**DOI:** 10.3389/fimmu.2026.1969351

**Published:** 2026-09-28

**Authors:** Ziheng Zheng, Xuechun Yang, Minqian Ou, Yuedong Chen, Deyong Gan

**Affiliations:** 1 The First Clinical College of Chongqing Medical University, Chongqing, China; 2 Department of Gastrointestinal Surgery, Tongliang Hospital of the First Affiliated Hospital of Chongqing Medical University, Chongqing, China

**Keywords:** autophagy, fatty-acid oxidation, immunometabolism, mitochondria, mitophagy, persistence, tissue niche, tissue-resident memory T cells

## Abstract

Tissue-resident memory T (TRM) cells persist at barrier and parenchymal sites where oxygen, nutrients, and inflammatory signals fluctuate. Their durability therefore cannot be explained by tissue retention alone. Evidence from skin, liver, intestine, lung, tumors, and inflamed tissues supports a coupled program in which local nutrient capture, mitochondrial fuel selection, organelle dynamics, autophagy, mitophagy, and proteostatic responses sustain survival while preserving rapid effector competence. We propose “organelle-niche matching” as a testable four-step working model: tissue inputs engage nutrient-, oxygen-, danger-, cytokine-, and antigen-sensing pathways; these regulate mitochondrial biogenesis, fusion-fission balance, respiratory-chain organization, and selective turnover; organelle state controls bioenergetic reserve, redox balance, apoptosis, and metabolite availability; and these outputs may reinforce or destabilize transcriptional residency and recall. The model integrates evidence across contexts, not one pathway established in a single TRM population. Direct TRM evidence is distinguished from tumor-infiltrating lymphocyte data and extrapolation from conventional memory CD8+ T cells. Lipid uptake and fatty-acid oxidation are prominent in selected TRM niches but are not universal, and mitophagy should not be equated with mitochondrial health. This framework identifies causal links already demonstrated in TRM cells, relationships that remain correlative, and therapeutic opportunities to strengthen protective residents without sustaining pathogenic populations.

## 1 Introduction

Tissue-resident memory T (TRM) cells are non-recirculating or slowly exchanging memory lymphocytes positioned in non-lymphoid and lymphoid tissues (1, 2). Early migration studies showed that memory T cells preferentially populate non-lymphoid sites (3), and skin infection models established that locally retained cells provide accelerated protection at portals of pathogen entry (4). Residency is actively imposed: suppression of S1PR1-dependent egress (5), tissue-dependent acquisition of CD103 (6), and transcriptional control by Hobit/Blimp-1 and Runx3 (7, 8) distinguish TRM cells from circulating memory. Human studies nevertheless reveal substantial phenotypic diversity rather than one invariant CD69+CD103+ state (9), while spatially discrete niches can instruct different functions within the same organ (10).

This heterogeneity creates a metabolic problem. Long-lived sentinels must survive nutrient limitation and intermittent hypoxia without sacrificing rapid cytokine production, cytotoxicity, or proliferative recall. We use “organelle-niche matching” to describe how local inputs are coupled to measurable changes in mitochondrial and lysosomal state and function. Here, organelle remodeling refers specifically to changes in organelle abundance, structure, composition, localization, or turnover—including mitochondrial biogenesis, fusion-fission and cristae/respiratory-chain organization, and autophagosome-lysosome-mediated selective clearance—that alter cellular function. We use metabolic adaptation more broadly to encompass changes in substrate uptake and use, bioenergetic and redox state, and extracellular metabolite sensing. These processes interact but are not synonymous. Organelle-niche matching is therefore proposed as an integrative mechanistic hypothesis, not an established universal pathway. To prevent evidence from different T-cell populations being conflated, we classify evidence throughout this review as: A, direct evidence in bona fide TRM cells; B, evidence in tumor-resident or tumor-infiltrating lymphocyte (TIL) populations with TRM-like features; and C, extrapolation from conventional circulating, splenic, central-memory, or effector-memory CD8+ T cells. Studies of other tumor-microenvironment cells are labeled contextual and are not used to infer a TRM-intrinsic mechanism (**Table 1**). Recent cancer syntheses likewise emphasize variable definitions of residence and the need for mechanistic validation (11, 12).

**Table 1:** Evidence hierarchy used to evaluate organelle-metabolic mechanisms.

Class	Experimental source	What the evidence can support	Main limitation
A: direct TRM	Tissue cells with residency established by location, retention/non-recirculation criteria, fate mapping, or a TRM-selective perturbation	A mechanism operating in the tested TRM population; causal only when directly perturbed	May remain tissue-, species-, or disease-specific
B: TRM-like tumor population	TIL or tumor-resident cells defined by CD69/CD103/CD49a, a residency signature, or spatial retention	A mechanism relevant to tumor-resident or TRM-like cells	Residency, exhaustion, and activation programs can overlap; recirculation may not be excluded
C: conventional memory CD8+ T cell	Circulating, splenic, central-memory, effector-memory, or *in-vitro*-derived memory cells	A mechanistic prior that generates a TRM hypothesis	Does not demonstrate that the pathway maintains established TRM cells
Contextual TME evidence	Dendritic cells, macrophages, natural killer cells, stromal cells, or tumor cells	How the niche may alter metabolites, cytokines, oxygen, or mitochondrial stress	Cannot establish a TRM-intrinsic pathway

## 2 Lipid access is a niche-dependent survival strategy

The clearest direct link between nutrient acquisition and TRM persistence comes from skin. Murine and human skin TRM cells express FABP4 and FABP5, acquire exogenous free fatty acids, and use mitochondrial β-oxidation; T-cell-specific loss of these transport proteins or inhibition of fatty-acid oxidation compromises long-term survival and antiviral protection (class A, causal) (13). This study established a direct, tissue-specific link between fatty-acid acquisition and TRM persistence, but it did not show that every tissue uses the same fuel. Bhlhe40 provides a second causal bridge: loss of this transcription factor impairs mitochondrial fitness, survival, and function in lung TRM cells and TILs (classes A and B, causal) (14). Hypoxia can induce elements of a human TRM differentiation program (class A, induction model), but its role in maintaining established human TRM cells remains unresolved (15).

“TRM cells burn fat” is therefore too broad. Fatty-acid oxidation supports CD8+ TRM-like survival in gastric adenocarcinoma (class B) (16), whereas it promotes apoptosis resistance and inflammatory behavior in Crohn’s disease CD4+ TRM cells (class A, disease-specific) (17). Fatty acids can invigorate tumor-resident responses (18), and conventional T-cell subsets use lipid metabolism as a salvage pathway when nutrients are poor (class C) (19). Integrin-defined murine memory subsets also retain distinct metabolic profiles (class C) (20). These findings support tissue- and lineage-specific lipid dependence rather than a universal TRM program, consistent with earlier syntheses of TRM metabolic longevity (21).

Substrate availability, intracellular transport, and respiratory capacity jointly determine the outcome. Lipid-rich skin, intestinal mucosa, inflamed synovium, and glucose-depleted tumors expose residents to different substrates. Increased lipid import without adequate electron transport or antioxidant buffering may cause lipotoxicity and mitochondrial reactive oxygen species (mtROS). Direct isotope tracing and maintenance-stage loss-of-function experiments remain scarce in human tissues; FABP or oxidation-enzyme transcripts alone do not demonstrate metabolic flux. Throughout this review, transcript or protein abundance is treated as evidence of pathway availability, functional uptake or oxidation assays as evidence of pathway use, isotope tracing as direct flux evidence, and perturbation as causal only when it changes a defined TRM outcome.

## 3 Working model of organelle-niche matching

This integrative working model contains four linked steps assembled from evidence obtained across different tissues, cell states, and experimental systems (**Figure 1**). Throughout this review, generation denotes differentiation toward a resident fate, establishment denotes tissue entry and acquisition of residency features, and persistence denotes maintenance after residency has been established. Step 1: niche input. Fatty acids, oxygen tension, extracellular ATP, TGF-β, IL-15 and other cytokines, costimulation, and antigen define the local energetic and stress landscape. Step 2: sensing and organelle remodeling. FABPs route lipid, hypoxia-responsive pathways report oxygen availability, P2RX7 detects extracellular ATP, and nutrient-sensitive mTOR/AMPK pathways influence the balance between biosynthesis and recycling. These signals are predicted to converge on mitochondrial biogenesis, fusion-fission, respiratory-chain organization, proteostasis, and selective turnover. Step 3: organelle output. The resulting mitochondrial pool determines spare respiratory capacity, membrane potential, mtROS, apoptotic threshold, and the supply of acetyl-CoA and other metabolites. Step 4: cell-state feedback. Bioenergetic and metabolite outputs can support chromatin accessibility, survival, and recall, whereas persistent depolarization, ROS, or respiratory failure can destabilize function or promote exhaustion.

**Figure 1:**
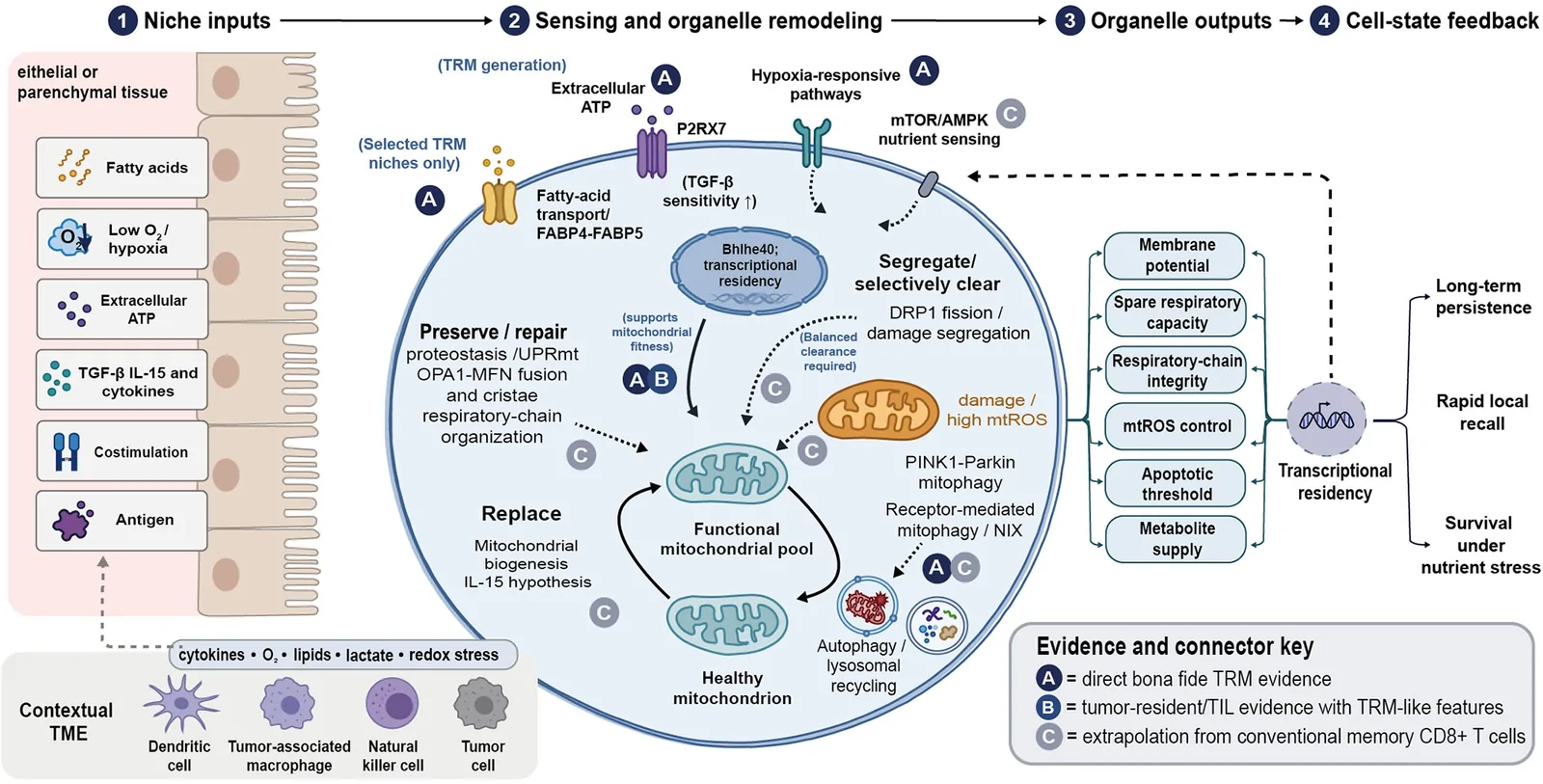
A working model of organelle-niche matching in tissue-resident memory T cell persistence. The model is organized from niche inputs, through sensing and organelle remodeling, to organelle and cell-state outputs. It integrates relationships demonstrated in different tissues, cell states, and experimental systems and should not be interpreted as a single pathway established within one TRM population. Local fatty acids are routed through FABP4/5 in selected niches; oxygen tension engages hypoxia-responsive pathways; extracellular ATP signals through P2RX7; and TGF-β and other cytokines, costimulatory ligands, and antigen signal through their cognate receptors, converging with nutrient- and activation-sensitive pathways. Collectively, these inputs may influence mitochondrial biogenesis, OPA1/mitofusin-dependent fusion, DRP1-dependent fission, respiratory-chain organization, proteostasis/UPRmt, PINK1-Parkin- or receptor-mediated mitophagy, and lysosomal recycling. The resulting mitochondrial pool shapes membrane potential, spare respiratory capacity, mtROS, apoptotic threshold, and metabolite supply, which may reinforce or destabilize transcriptional residency, persistence, and recall. Evidence badges are attached to individual nodes or connections: A indicates direct evidence in bona fide TRM cells; B, evidence in tumor-resident or TIL populations with TRM-like features; and C, extrapolation from conventional memory CD8+ T cells. These badges classify the experimental evidence and do not imply that every mechanism has been demonstrated during long-term TRM maintenance; evidence concerning generation, establishment, or persistence is specified in the main text. Contextual evidence from other tumor-microenvironment cells is displayed separately and is not treated as proof of a TRM-intrinsic mechanism.

The causal strength and biological stage of individual links differ. P2RX7 directs metabolic fitness in long-lived conventional memory CD8+ T cells (class C; memory-formation evidence) (22) and promotes TRM generation by increasing TGF-β sensitivity (class A, causal; generation-stage evidence) (23), while P2RX7 imprinting of a pro-memory program in effector CD8+ T cells remains class C, formation-stage evidence (24). Bhlhe40 directly links a residency-associated transcription factor to mitochondrial and epigenetic fitness in lung TRM cells (class A, causal; persistence/fitness evidence) (14). In contrast, the proposition that mitochondrial metabolites continuously maintain the full TRM transcriptional program is plausible but has not been demonstrated by maintenance-stage perturbation.

Turnover and biogenesis should be treated as distinct decisions. In conventional memory CD8+ T cells, IL-15 increases mitochondrial biogenesis and spare respiratory capacity (class C, causal) (25). This provides a testable TRM hypothesis but cannot be assumed to operate in every resident population. By comparison, mitochondrial damage, loss of membrane potential, or proteotoxic stress may favor repair, fission-mediated segregation, or mitophagy. Which niche signals select replacement rather than clearance in established TRM cells is unknown. A decisive experiment would induce pathway deletion only after residence is established, then quantify mitochondrial mass, respiratory-chain integrity, flux, survival, and local recall in the same cells.

## 4 Mitochondrial quality control is a balance, not a synonym for mitophagy

Long-term tissue residence creates an organelle-maintenance challenge. Human liver memory CD8+ T cells use autophagy to support tissue residence (class A, evidence in resident cells) (26); however, this study does not by itself establish a universal maintenance mechanism across TRM populations. By contrast, the requirement for autophagy during conventional CD8+ T-cell memory formation (27, 28) and the roles of NIX-mediated or broader mitophagy circuits during effector-memory formation (29, 30) are class C, formation-stage evidence. These studies provide mechanistic priors for TRM research but do not demonstrate maintenance of established TRM cells. In rheumatoid arthritis, mitophagy and mitochondrial control of proteasomal programs are associated with abnormal TRM differentiation (class A, human disease evidence) (31, 32), but cross-sectional inflammatory tissue cannot establish maintenance-stage causality.

Mitochondrial quality control comprises at least five coordinated modules. First, proteostasis and stress signaling use mitochondrial chaperones, proteases, the ubiquitin-proteasome system, and the mitochondrial unfolded-protein response (UPRmt) to repair or contain damage before whole-organelle removal (33). Second, fusion mediated by OPA1 and mitofusins permits content mixing and can preserve cristae organization. In conventional memory CD8+ T cells, OPA1-dependent fusion supports electron-transport-chain supercomplex organization and oxidative metabolism (class C, causal) (34). Third, fission mediated by DRP1 segregates damaged regions but also controls activation and population fitness; DRP1 deletion can favor memory formation in isolation yet impair competitive memory responses (class C, causal) (35). Fourth, selective mitophagy removes organelles that cannot be repaired (36). PINK1-Parkin-dependent mitophagy senses sustained membrane-potential loss, whereas receptor-mediated routes, including NIX-dependent pathways, can operate without Parkin. As contextual tumor-cell evidence, a drug-combination strategy that impaired DRP1-mediated fission in PINK1-low gastric adenocarcinoma cells induced mitochondrial pathology and cell death (37); this finding illustrates the therapeutic manipulability of mitochondrial quality-control imbalance in tumor cells but does not establish a TRM-intrinsic mechanism. Neither PINK1-Parkin-dependent nor receptor-mediated mitophagy has yet been shown to universally maintain bona fide TRM cells. Fifth, biogenesis replenishes the network after clearance. These modules form an interdependent surveillance system rather than a linear “more mitophagy is better” pathway. Except where an A or B designation is specified, this five-module architecture synthesizes class C or general cell-biological evidence and should be tested directly in established TRM populations.

The relevant endpoint is a functionally competent mitochondrial pool. Insufficient repair or clearance permits depolarized mitochondria, respiratory-chain defects, excess mtROS, and apoptosis. Excessive fission and clearance can reduce mitochondrial mass and spare respiratory capacity, while unopposed fusion can retain damaged material. Measurements should therefore combine mitophagic flux with membrane potential, mitochondrial mass, cristae morphology, complex I-V activity or respiratory supercomplex organization, mtROS, ATP-linked respiration, and recall function. Static abundance of LC3, PINK1, Parkin, NIX, or mitochondrial transcripts cannot distinguish active flux from a blocked pathway.

## 5 Metabolic communication and the residency circuit

Metabolic adaptation is embedded in a broader residency network, but the available studies address different biological stages. mTOR regulates conventional memory CD8+ T-cell differentiation (class C; formation-stage evidence) (38), whereas 4-1BB signaling promotes lung TRM formation through a TRAF1-, mTOR-, and antigen-dependent mechanism (class A; generation-stage evidence) (39). Tissue ICOS engagement supports CD8+ TRM establishment (40), dopamine signaling enhances resident differentiation (41), and retinoic acid acts during priming to license intestinal CD103+ TRM development (42); these are generation- or establishment-stage findings rather than tests of long-term maintenance. Whether any of these signals continuously supports established TRM cells remains to be determined.

In tumors, “niche” is partly constructed by neighboring cells. Dendritic cells, tumor-associated macrophages, and natural killer cells undergo their own mitochondrial and immunometabolic remodeling (43–45). These contextual studies show how competing cells may alter cytokines, oxygen, lipids, lactate, and redox stress, but they do not demonstrate metabolite transfer to TRM cells. Direct communication should be tested using spatial metabolomics, isotope tracing across defined cell types, or cell-specific transporter perturbation. Tumor-resident memory cells correlate with favorable outcomes, yet marker-defined TILs may combine residency, activation, and exhaustion programs (11, 12).

Retention and metabolic exposure also reinforce each other. CD49a identifies human skin TRM states poised for cytotoxicity (46), and S1PR5 regulates peripheral retention of resident lymphocytes (47). Remaining in one site preserves access to local lipid and cytokine sources but also prolongs exposure to hypoxia, ATP, and antigen. Organelle remodeling may therefore be both a cause and a consequence of residence.

## 6 Protection, pathology, and therapeutic selectivity

Protection requires more than persistence. Airway and lung-interstitial TRM subsets mount rapid antiviral responses (48, 49), bystander activation can recruit neutrophils during bacterial pneumonia (50), and human lung-resident responses are detectable after SARS-CoV-2 infection and experimental influenza (51, 52). HIV studies further show that tissue-resident compartments may be depleted or retain function during inflammation (53, 54). These studies establish functional heterogeneity but largely do not test organelle or metabolic mechanisms; here, they define the protective outcomes that a proposed metabolic intervention would need to preserve or improve.

In chronic inflammation, JAK/STAT-associated renal TRM fate, IL-17A-producing skin residents, Candida-induced TH17 residents, and intestinal or joint TRM subsets illustrate tissue- and disease-specific TRM states (55–63). Most of these studies describe phenotype, localization, transcriptional state, or disease association rather than direct metabolic flux or organelle perturbation. They therefore show why organelle-metabolic programs must be evaluated against pathological function, not that one metabolic pathway drives all inflammatory diseases.

Tumors combine chronic antigen exposure, nutrient competition, and suppressive signaling. Tumor-resident HBV-specific cells correlate with relapse-free survival (64), CXCR6 supports retention or recruitment and tumor control (65, 66), and CD103+CD8+ infiltration is associated with clinical outcome (67). These findings support relationships involving resident abundance, retention, or outcome, but they do not establish a general metabolic therapy. Mechanistic studies must determine whether an intervention restores respiratory or lysosomal competence in protective residents or instead sustains dysfunctional TILs or pathogenic residents.

## 7 Discussion

Organelle-niche matching is best viewed as an integrative, hypothesis-generating framework rather than an experimentally established universal pathway. Its strongest causal anchors are tissue-specific: FABP4/5-dependent lipid use in skin TRM cells, Bhlhe40-dependent mitochondrial fitness in lung TRM cells, and P2RX7-TGF-β coupling during TRM generation. Evidence for OPA1-dependent fusion, DRP1-dependent fission, IL-15-driven biogenesis, NIX-mediated mitophagy, PINK1-Parkin surveillance, and UPRmt is mechanistically valuable but remains largely class C or general cell-biological evidence. The revised evidence hierarchy makes these boundaries explicit.

Four priorities follow. First, metabolic flux should be measured directly rather than inferred from RNA or protein abundance. Second, inducible perturbations after TRM establishment are required to distinguish formation from maintenance. Third, mitochondrial morphology, cristae organization, membrane potential, respiratory-chain function, mtROS, mitophagic flux, and lysosomal competence should be measured together in intact tissue. Fourth, protective and pathogenic TRM cells should be compared within the same organ while accounting for age, sex, diet, microbiota, antigen persistence, and therapy.

These therapeutic implications should be regarded as experimental opportunities rather than clinically actionable strategies. In preclinical studies, vaccination could be used to test whether transiently improving precursor mitochondrial fitness or tissue lipid access enhances seeding. Studies of established antitumor TRM cells could test restoration of a defined mitochondrial or lysosomal quality-control defect, whereas studies of pathogenic TRM cells could test disruption of a tissue-specific fuel dependency. Modulation of P2RX7-TGF-β signaling likewise requires stage-specific efficacy and safety testing before therapeutic interpretation. Each proposal requires direct functional endpoints and validation in human tissue; abundance, residency markers, mitochondrial mass, or mitophagy markers alone are inadequate surrogates. These proposals should not be interpreted as clinical recommendations.

The principal limitation is uneven causal depth. Human evidence is often cross-sectional, tissue dissociation perturbs metabolites and organelle stress, and TILs cannot automatically be equated with bona fide TRM cells. “Organelle-niche matching” therefore identifies the experiments needed to convert a compelling correlation into a mechanism: define the resident population, perturb a specific niche sensor or organelle process after residence is established, verify organelle function, and test local recall. This discipline should make it possible to fuel beneficial sentinels without inadvertently sustaining chronic inflammatory disease.
